# (*E*)-6-(Furan-2-yl­methyl­idene)-6,7,8,9-tetra­hydro­pyrido[2,1-*b*]quinazoline-11-thione

**DOI:** 10.1107/S2414314620003569

**Published:** 2020-03-13

**Authors:** Akmal Tojiboev, Azizbek Nasrullaev, Kambarali Turgunov, Burkhan Elmuradov, Bakhodir Tashkhodjaev

**Affiliations:** a Institute of Ion-Plasma and Laser Technologies, Academy of Sciences of Uzbekistan, Durmon Yuli Str. 33, 100125 Tashkent, Uzbekistan; b S. Yunusov Institute of Chemistry of Plant Substances, Academy of Sciences of Uzbekistan, Mirzo Ulugbek Str. 77, 100170 Tashkent, Uzbekistan; c Turin Polytechnic University in Tashkent, Kichik Khalka Yuli Str. 17, Tashkent 100095, Uzbekistan; Benemérita Universidad Autónoma de Puebla, México

**Keywords:** crystal structure, quinazolinthione, *E*-configuration

## Abstract

A quinazolinthione, C_17_H_14_N_2_OS, was synthesized by the condensation reaction of 6,7,8,9-tetra­hydro-11*H*-pyrido[2,1-*b*]quinazolin-11-thione with furfural. The mol­ecule crystallizes in the monoclinic system (*Cc* space group) and has an *E* configuration with respect to the exocyclic C=C bond. In the crystal, mol­ecules are linked through C—H⋯π(furan) inter­actions, forming zigzag chains propagating along the [001] direction.

## Abstract 2

A new quinazolinthione derivative was synthesized, and X-ray diffraction analysis proved the *E* configuration for the exocyclic double bond in the mol­ecule.

## Graphical abstract

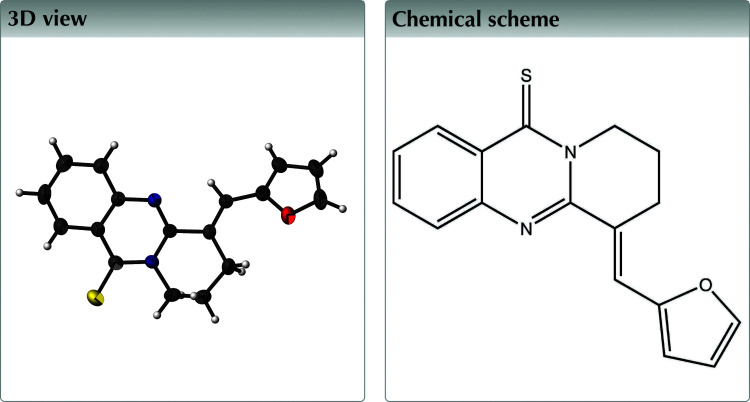

## Structure description

Quinazoline derivatives are biologically active heterocyclic compounds (Shakhidoyatov, 1988 ▸; Elmuradov & Shakhidoyatov, 2006 ▸), used as drugs, such as cardiovascular agents (Volzhina & Yakhontov, 1982 ▸), herbicides (Chupp, 1974 ▸; Dayan, 2019 ▸), fungicides (Vicentini *et al.*, 2002 ▸; Sun *et al.*, 2011 ▸), *etc*. Among them, quinazoline and its homologues exhibit plural reactivity while maintaining several functional groups. The study of their reaction properties is of theoretical inter­est (Shakhidoyatov & Elmuradov, 2014 ▸). Alkyl­ation and condensation reactions have been previously studied to produce tricyclic derivatives of quinazolinthione (Nasrullayev *et al.*, 2012 ▸; Nasrullaev *et al.*, 2015 ▸, 2016 ▸, 2017 ▸). In the present work, we report the crystal structure of a new quinazolinthione derivative.

**Figure 1:**
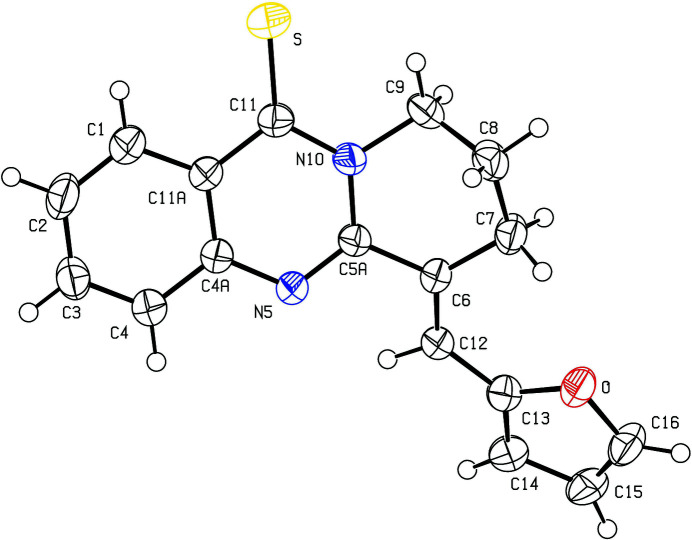
The mol­ecular structure of title compound with the atom labelling. Displacement ellipsoids are drawn at the 50% probability level.

The title compound (Fig. 1 ▸), consist of 6,7,8,9-tetra­hydro­pyrido[2,1-*b*]quinazoline and furan-2-yl­methyl­ene groups linked through the C6=C12 double bond [1.348 (5) Å]. The mol­ecule adopts an *E* configuration relative to this bond. The quinazoline moiety is almost planar with anr.m.s. deviation of 0.0234 Å. Atoms C7 and C8 deviate from the plane through atoms C6, C5*A*, N10, C9 (r.m.s. deviation of 0.0053 Å) of the six-membered tetra­methyl­ene ring by 0.418 (8) and 0.912 (9) Å, respectively. These values are similar to those found for related compounds, for example 6,7,8,9-tetra­hydro-11*H*-pyrido[2,1-*b*]quinazolin-11-thione and 6,7,8,9,10,12-hexa­hydro­azepino[2,1-*b*]quinazolin-12-thione (Nasrullayev *et al.*, 2016 ▸).

**Figure 2:**
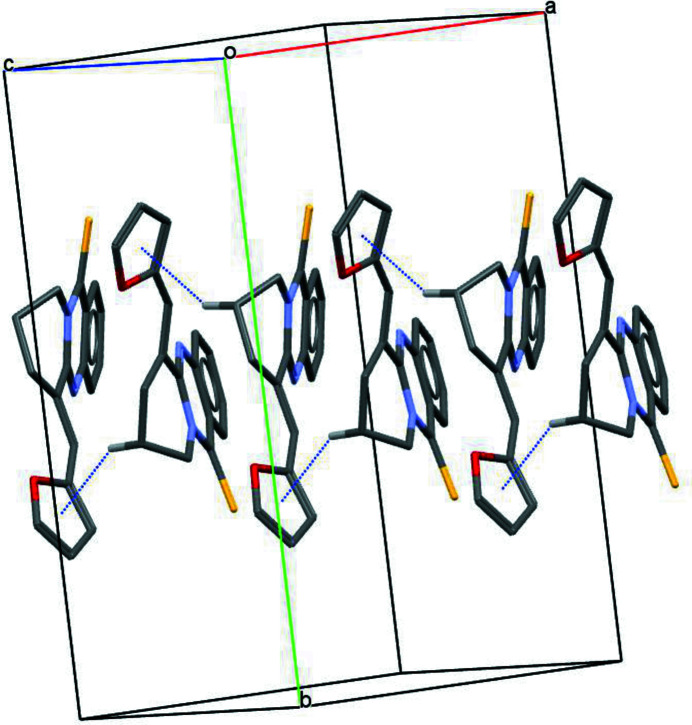
Chain of mol­ecules of the title compound linked by C—H⋯π inter­actions. For clarity, H atoms not involved in these inter­actions have been omitted, and only atom H8*B* has been included.

In the crystal, mol­ecules are linked by C—H⋯π(furan) inter­actions between mol­ecules related by the *c* glide plane of space group *Cc*, forming zigzag chains propagating along the [001] direction (Table 1 ▸, Fig. 2 ▸).

## Synthesis and crystallization

6,7,8,9-Tetra­hydro-11*H*-pyrido[2,1-*b*]quinazolin-11-thione (1 mmol) was dissolved in 2–3 ml of glacial acetic acid and furfural (1 mmol) was added. The reaction mixture was refluxed for 5.5 h and cooled. Distilled water (10 ml) was added to the reaction mixture and the precipitate that formed was filtered off, washed with distilled water and dried. After recrystallization from cyclo­hexa­ne solution, the title compound was recovered in good yield (68%), m.p. 170°C, *R*_f_ = 0.88. ^1^H NMR, δ, p.p.m., *J* (Hz): 8.28 (1*H*, *d*, *J* = 8.2, H-1), 7.5 (1*H*, *t*, *J* = 8.2, H-2), 7.39 (1*H*, *d*, *J* = 1.7, H-5′), 7.30 (1*H*, *t*, *J* = 1.7, =CH), 7.23–7.29 (2*H*, *m*, H-3,4), 6.68 (1*H*, *d*, *J* = 3.4, H-3′), 6.3 (1*H*, *dd*, *J* = 3.4, *J* = 1.7, H-4′), 4.36 (2*H*, *t*, *J* = 5.5, δ-CH_2_), 2.78 (2*H*, *dt*, *J* = 6.8, *J* = 1.7, β-CH_2_), 1.85 (2*H*, *m*, γ-CH_2_). IR spectrum: ν, cm^−1^: 1569 (C=N), 1469 (C—N), 1272 (C=S). Light-orange prismatic single crystals suitable for X-ray diffraction analysis were obtained by were grown from acetone by slow evaporation of the solvent.

## Refinement

Crystal data, data collection and structure refinement details are summarized in Table 2 ▸.

## Supplementary Material

Crystal structure: contains datablock(s) I. DOI: 10.1107/S2414314620003569/bh4051sup1.cif

Structure factors: contains datablock(s) I. DOI: 10.1107/S2414314620003569/bh4051Isup2.hkl

Supporting information file. DOI: 10.1107/S2414314620003569/bh4051Isup3.cml

CCDC reference: 1989156

Additional supporting information:  crystallographic information; 3D view; checkCIF report

## Figures and Tables

**Table 1:** Hydrogen-bond geometry (Å, °) *Cg* is the centroid of the furan ring (O/C2–C16).

*D*—H⋯*A*	*D*—H	H⋯*A*	*D*⋯*A*	*D*—H⋯*A*
C8—H8*B*⋯*Cg* ^i^	0.97	2.76	3.596 (5)	145

Symmetry code: (i) 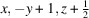 .

**Table 2:** Experimental details

Crystal data	
Chemical formula	C_17_H_14_N_2_OS
*M*_r_	294.36
Crystal system, space group	Monoclinic, *C* *c*
Temperature (K)	295
*a*, *b*, *c* (Å)	9.4340 (19), 17.134 (4), 8.8260 (18)
β (°)	105.01 (4)
*V* (Å^3^)	1378.0 (6)
*Z*	4
Radiation type	Cu *K*α
μ (mm^−1^)	2.08
Crystal size (mm)	0.50 × 0.20 × 0.20

Data collection	
Diffractometer	Oxford Diffraction Xcalibur, Ruby
Absorption correction	Multi-scan (*CrysAlis PRO*; Oxford Diffraction, 2009 ▸)
*T*_min_, *T*_max_	0.371, 1.000
No. of measured, independent and observed [*I* > 2σ(*I*)] reflections	2724, 1969, 1717
*R*_int_	0.028
(sin θ/λ)_max_ (Å^−1^)	0.628

Refinement	
*R*[*F* ^2^ > 2σ(*F* ^2^)], *wR*(*F* ^2^), *S*	0.045, 0.123, 1.04
No. of reflections	1969
No. of parameters	190
No. of restraints	2
H-atom treatment	H-atom parameters constrained
Δρ_max_, Δρ_min_ (e Å^−3^)	0.22, −0.25
Absolute structure	Flack *x* determined using 451 quotients [(*I* ^+^)−(*I* ^−^)]/[(*I* ^+^)+(*I* ^−^)] (Parsons *et al.*, 2013 ▸)
Absolute structure parameter	0.12 (3)

Computer programs: *CrysAlis PRO* (Oxford Diffraction, 2009 ▸), *SHELXS97* (Sheldrick, 2008 ▸), *SHELXL2014* (Sheldrick, 2015 ▸), *PLATON* (Spek, 2020 ▸) and *publCIF* (Westrip, 2010 ▸).

## full crystallographic data

### Crystal data

**Table:**

C_17_H_14_N_2_OS	*D*_x_ = 1.419 Mg m^−^^3^
*M**_r_* = 294.36	Melting point: 443 K
Monoclinic, *C**c*	Cu *K*α radiation, λ = 1.54184 Å
*a* = 9.4340 (19) Å	Cell parameters from 1371 reflections
*b* = 17.134 (4) Å	θ = 5.2–75.6°
*c* = 8.8260 (18) Å	µ = 2.08 mm^−^^1^
β = 105.01 (4)°	*T* = 295 K
*V* = 1378.0 (6) Å^3^	Prism, light-orange
*Z* = 4	0.50 × 0.20 × 0.20 mm
*F*(000) = 616	

### Data collection

**Table:**

Oxford Diffraction Xcalibur, Ruby diffractometer	1969 independent reflections
Radiation source: Enhance (Cu) X-ray Source	1717 reflections with *I* > 2σ(*I*)
Detector resolution: 10.2576 pixels mm^-1^	*R*_int_ = 0.028
ω scans	θ_max_ = 75.7°, θ_min_ = 5.2°
Absorption correction: multi-scan (CrysAlis PRO; Oxford Diffraction, 2009)	*h* = −11→11
*T*_min_ = 0.371, *T*_max_ = 1.000	*k* = −21→20
2724 measured reflections	*l* = −10→8

### Refinement

**Table:**

Refinement on *F*^2^	Hydrogen site location: inferred from neighbouring sites
Least-squares matrix: full	H-atom parameters constrained
*R*[*F*^2^ > 2σ(*F*^2^)] = 0.045	*w* = 1/[σ^2^(*F*_o_^2^) + (0.0857*P*)^2^] where *P* = (*F*_o_^2^ + 2*F*_c_^2^)/3
*wR*(*F*^2^) = 0.123	(Δ/σ)_max_ < 0.001
*S* = 1.03	Δρ_max_ = 0.22 e Å^−^^3^
1969 reflections	Δρ_min_ = −0.25 e Å^−^^3^
190 parameters	Absolute structure: Flack *x* determined using 451 quotients [(*I*^+^)-(*I*^-^)]/[(*I*^+^)+(*I*^-^)] (Parsons *et al.*, 2013)
2 restraints	Absolute structure parameter: 0.12 (3)

### Special details

**Table:**

Refinement. All C-bound H atoms were positioned geometrically and refined using a riding model, with C—H = 0.93–0.97 Å and *U*_iso_(H) = 1.2*U*_eq_(carrier C).

### Fractional atomic coordinates and isotropic or equivalent isotropic displacement parameters (Å^2^)

**Table:**

	*x*	*y*	*z*	*U*_iso_*/*U*_eq_	
S	0.84058 (15)	0.72703 (6)	0.43163 (16)	0.0570 (4)	
O	0.3377 (3)	0.35652 (18)	0.0790 (4)	0.0470 (7)	
N5	0.7682 (4)	0.46996 (18)	0.4826 (4)	0.0375 (7)	
C5A	0.6819 (4)	0.5126 (2)	0.3748 (4)	0.0335 (8)	
N10	0.6960 (4)	0.59229 (18)	0.3632 (4)	0.0361 (7)	
C11	0.8105 (4)	0.6337 (2)	0.4601 (5)	0.0371 (9)	
C11A	0.9040 (4)	0.5873 (2)	0.5853 (5)	0.0348 (8)	
C1	1.0187 (5)	0.6216 (2)	0.7004 (6)	0.0458 (10)	
H1A	1.0354	0.6750	0.6982	0.055*	
C2	1.1058 (5)	0.5765 (3)	0.8156 (6)	0.0523 (11)	
H2A	1.1800	0.5998	0.8926	0.063*	
C3	1.0842 (5)	0.4955 (3)	0.8188 (6)	0.0474 (10)	
H3A	1.1453	0.4652	0.8963	0.057*	
C4	0.9728 (4)	0.4610 (2)	0.7072 (5)	0.0417 (9)	
H4A	0.9585	0.4073	0.7095	0.050*	
C4A	0.8803 (4)	0.5065 (2)	0.5895 (5)	0.0352 (8)	
C6	0.5610 (4)	0.4721 (2)	0.2591 (5)	0.0347 (8)	
C7	0.4301 (5)	0.5176 (2)	0.1681 (5)	0.0429 (10)	
H7A	0.3412	0.4939	0.1828	0.051*	
H7B	0.4261	0.5153	0.0573	0.051*	
C8	0.4371 (5)	0.6018 (2)	0.2199 (6)	0.0478 (11)	
H8A	0.3663	0.6321	0.1432	0.057*	
H8B	0.4115	0.6053	0.3193	0.057*	
C9	0.5873 (5)	0.6349 (2)	0.2377 (6)	0.0493 (11)	
H9A	0.6143	0.6303	0.1392	0.059*	
H9B	0.5876	0.6898	0.2643	0.059*	
C12	0.5812 (4)	0.3956 (2)	0.2360 (5)	0.0376 (9)	
H12A	0.6717	0.3757	0.2907	0.045*	
C13	0.4845 (5)	0.3402 (2)	0.1402 (5)	0.0391 (9)	
C14	0.5114 (6)	0.2665 (2)	0.0973 (6)	0.0481 (11)	
H14A	0.6018	0.2413	0.1226	0.058*	
C15	0.3780 (6)	0.2352 (3)	0.0079 (6)	0.0539 (12)	
H15A	0.3628	0.1856	−0.0360	0.065*	
C16	0.2774 (6)	0.2914 (3)	−0.0014 (6)	0.0536 (12)	
H16A	0.1791	0.2867	−0.0556	0.064*	

### Atomic displacement parameters (Å^2^)

**Table:**

	*U*^11^	*U*^22^	*U*^33^	*U*^12^	*U*^13^	*U*^23^
S	0.0620 (7)	0.0336 (5)	0.0682 (8)	−0.0066 (5)	0.0039 (6)	0.0005 (5)
O	0.0388 (14)	0.0429 (16)	0.0531 (19)	−0.0076 (12)	0.0006 (14)	−0.0058 (14)
N5	0.0360 (15)	0.0295 (15)	0.0408 (19)	−0.0022 (13)	−0.0013 (14)	0.0014 (13)
C5A	0.0355 (18)	0.0289 (17)	0.033 (2)	0.0003 (15)	0.0039 (16)	0.0005 (15)
N10	0.0420 (17)	0.0263 (15)	0.0349 (18)	0.0003 (13)	0.0007 (15)	−0.0001 (13)
C11	0.038 (2)	0.0310 (17)	0.041 (2)	0.0002 (15)	0.0078 (17)	−0.0006 (15)
C11A	0.0349 (17)	0.0320 (17)	0.036 (2)	−0.0028 (15)	0.0058 (17)	−0.0019 (15)
C1	0.044 (2)	0.040 (2)	0.048 (3)	−0.0052 (17)	0.002 (2)	−0.0046 (18)
C2	0.041 (2)	0.060 (3)	0.048 (3)	−0.011 (2)	−0.004 (2)	−0.009 (2)
C3	0.038 (2)	0.056 (3)	0.043 (2)	0.0028 (18)	0.0003 (19)	0.005 (2)
C4	0.038 (2)	0.0384 (19)	0.044 (3)	−0.0003 (16)	0.0030 (19)	0.0025 (17)
C4A	0.0320 (17)	0.0336 (17)	0.037 (2)	−0.0013 (14)	0.0040 (17)	0.0006 (15)
C6	0.0319 (17)	0.0336 (18)	0.035 (2)	−0.0034 (15)	0.0017 (16)	−0.0014 (14)
C7	0.0366 (19)	0.041 (2)	0.044 (2)	−0.0005 (16)	−0.0024 (18)	−0.0082 (17)
C8	0.047 (2)	0.037 (2)	0.051 (3)	0.0117 (17)	−0.003 (2)	−0.0025 (18)
C9	0.057 (3)	0.035 (2)	0.046 (3)	0.0042 (19)	−0.005 (2)	0.0103 (18)
C12	0.0372 (18)	0.0307 (18)	0.040 (2)	−0.0014 (15)	0.0001 (17)	0.0028 (16)
C13	0.042 (2)	0.037 (2)	0.035 (2)	−0.0020 (16)	0.0048 (18)	−0.0004 (16)
C14	0.054 (2)	0.033 (2)	0.051 (3)	−0.0027 (18)	0.003 (2)	−0.0029 (18)
C15	0.067 (3)	0.035 (2)	0.054 (3)	−0.014 (2)	0.004 (2)	−0.0083 (19)
C16	0.050 (2)	0.053 (3)	0.051 (3)	−0.019 (2)	0.001 (2)	−0.010 (2)

### Geometric parameters (Å, º)

**Table:**

S—C11	1.655 (4)	C4—H4A	0.9300
O—C16	1.365 (5)	C6—C12	1.348 (5)
O—C13	1.378 (5)	C6—C7	1.504 (5)
N5—C5A	1.304 (5)	C7—C8	1.510 (5)
N5—C4A	1.373 (5)	C7—H7A	0.9700
C5A—N10	1.379 (4)	C7—H7B	0.9700
C5A—C6	1.491 (5)	C8—C9	1.497 (7)
N10—C11	1.387 (5)	C8—H8A	0.9700
N10—C9	1.491 (5)	C8—H8B	0.9700
C11—C11A	1.458 (6)	C9—H9A	0.9700
C11A—C4A	1.405 (5)	C9—H9B	0.9700
C11A—C1	1.406 (6)	C12—C13	1.431 (6)
C1—C2	1.368 (7)	C12—H12A	0.9300
C1—H1A	0.9300	C13—C14	1.361 (6)
C2—C3	1.404 (7)	C14—C15	1.407 (7)
C2—H2A	0.9300	C14—H14A	0.9300
C3—C4	1.374 (6)	C15—C16	1.340 (7)
C3—H3A	0.9300	C15—H15A	0.9300
C4—C4A	1.407 (5)	C16—H16A	0.9300
			
C16—O—C13	106.2 (4)	C6—C7—H7A	109.3
C5A—N5—C4A	118.1 (3)	C8—C7—H7A	109.3
N5—C5A—N10	123.7 (3)	C6—C7—H7B	109.3
N5—C5A—C6	117.5 (3)	C8—C7—H7B	109.3
N10—C5A—C6	118.8 (3)	H7A—C7—H7B	108.0
C5A—N10—C11	122.4 (3)	C9—C8—C7	111.1 (4)
C5A—N10—C9	118.7 (3)	C9—C8—H8A	109.4
C11—N10—C9	118.9 (3)	C7—C8—H8A	109.4
N10—C11—C11A	114.2 (3)	C9—C8—H8B	109.4
N10—C11—S	122.5 (3)	C7—C8—H8B	109.4
C11A—C11—S	123.2 (3)	H8A—C8—H8B	108.0
C4A—C11A—C1	119.3 (4)	N10—C9—C8	110.0 (4)
C4A—C11A—C11	119.2 (3)	N10—C9—H9A	109.7
C1—C11A—C11	121.4 (4)	C8—C9—H9A	109.7
C2—C1—C11A	120.2 (4)	N10—C9—H9B	109.7
C2—C1—H1A	119.9	C8—C9—H9B	109.7
C11A—C1—H1A	119.9	H9A—C9—H9B	108.2
C1—C2—C3	120.7 (4)	C6—C12—C13	129.8 (4)
C1—C2—H2A	119.7	C6—C12—H12A	115.1
C3—C2—H2A	119.7	C13—C12—H12A	115.1
C4—C3—C2	120.0 (4)	C14—C13—O	108.7 (4)
C4—C3—H3A	120.0	C14—C13—C12	130.0 (4)
C2—C3—H3A	120.0	O—C13—C12	121.3 (4)
C3—C4—C4A	120.2 (4)	C13—C14—C15	107.8 (4)
C3—C4—H4A	119.9	C13—C14—H14A	126.1
C4A—C4—H4A	119.9	C15—C14—H14A	126.1
N5—C4A—C11A	122.1 (3)	C16—C15—C14	106.1 (4)
N5—C4A—C4	118.3 (3)	C16—C15—H15A	127.0
C11A—C4A—C4	119.6 (4)	C14—C15—H15A	127.0
C12—C6—C5A	116.3 (3)	C15—C16—O	111.2 (4)
C12—C6—C7	123.5 (3)	C15—C16—H16A	124.4
C5A—C6—C7	120.1 (3)	O—C16—H16A	124.4
C6—C7—C8	111.6 (3)		

### Hydrogen-bond geometry (Å, º)

*Cg* is the centroid of the furan ring (O/C2–C16).

**Table:**

*D*—H···*A*	*D*—H	H···*A*	*D*···*A*	*D*—H···*A*
C8—H8*B*···*Cg*^i^	0.97	2.76	3.596 (5)	145

Symmetry code: (i) *x*, −*y*+1, *z*+1/2.
